# Measuring the Capacity to Love: Development of the CTL-Inventory

**DOI:** 10.3389/fpsyg.2018.01115

**Published:** 2018-07-24

**Authors:** Nestor D. Kapusta, Konrad S. Jankowski, Viktoria Wolf, Magalie Chéron-Le Guludec, Madlen Lopatka, Christopher Hammerer, Alina Schnieder, David Kealy, John S. Ogrodniczuk, Victor Blüml

**Affiliations:** ^1^Department for Psychoanalysis and Psychotherapy, Medical University of Vienna, Vienna, Austria; ^2^Faculty of Psychology, University of Warsaw, Warsaw, Poland; ^3^Department of Psychiatry, University of British Columbia, Vancouver, BC, Canada

**Keywords:** Capacity to Love Inventory (CTL-I), psychometrics properties, validity and reliability, psychotherapy, psychoanalytic theory

## Abstract

**Objective:** The individual capacity to love (CTL) has been linked to various mental health parameters and is considered to be an important outcome parameter of psychotherapeutic treatment. However, empirical examinations of the concept have not been conducted up to now. The aim of this study was to develop a valid and reliable instrument for the assessment of CTL [Capacity to Love Inventory (CTL-I)] as a trait of personality, which is shown to be related to clinically relevant symptoms and conditions.

**Method:** Four independent healthy samples in Austria (*n* = 547, *n* = 174, and *n* = 85) and Poland (*n* = 240) were assessed by a prototype of the CTL-I and its final shorter version in a confirmatory factor analysis (CFA). Internal consistency of the total questionnaire and each subscale was assessed by Cronbach alpha. External validity was measured against Beck Depression Inventory, Quality of Relationship Inventory, Sociosexual Orientation Inventory, Pathological Narcissism Inventory, and Narcissistic Personality Inventory according to the theoretical framework of the CTL concept. Further test–retest reliability was assessed.

**Results:** The CFA confirmed 41 items in six dimensions: Interest in the life project of the other, Basic trust, Humility and gratitude, Common ego ideal, Permanence of sexual passion, and Acceptance of loss/jealousy/mourning. The Cronbach alphas of the total CTL-I and its subscales ranged between 0.67 and 0.90 in all samples, suggesting a valid construct. The CTL-I was moderately positively associated with quality of relationship (Support *r* = 0.63, Conflict *r* = -0.66, and Depth *r* = 0.66) and inversely associated with symptoms of depression (*r* = -0.37), pathological narcissism (*r* = -0.29) and promiscuity (*r* = -0.42). The test–retest reliability of the total CTL-I was high with *r* = 0.81, suggesting the stability of answers over time.

**Conclusion:** The proposed 41-item version of the CTL-I is a psychometrically sound and validated instrument measuring six dimensions of the concept of the CTL. The reported negative associations with clinically relevant parameters such as depression, pathological narcissism and promiscuity as well as associations with relationship qualities such as conflicts, support, and depth warrant its future use in burdened populations including couples in clinical settings.

“…we must begin to love in order not to fall ill…”

Freud, 1914, p. 85

## Introduction

Love is one of the most fundamental human phenomena and it has been the subject of poets, philosophers and religious considerations for millennia. As an evolved commitment love is linked to better health and survival, and plays a critical role in the evolution of humans (Fletcher et al., 2015). Despite its ubiquity, research has not agreed upon a theoretical basis for the phenomenon of love, resulting in many open questions for research (Levin, 2000). Some of the first empirical psychological and sociological approaches focused on declared aspects of love such as its romantic features (Rubin, 1970) or attempted to define love-styles such as eros, agape, pragma, mania, storge, ludus (Lee, 1973, 1977; Hendrick and Hendrick, 1986). Following the scientific differentiation of love aspects and styles, the first attempt of an unified theory of love was established empirically by Sternberg (1986) with the introduction of love-components such as *intimacy*, *passion* and *commitment*, which facilitate description of different phenotypes of love relations. However, although these descriptive typologies helped to establish phenotypic love styles, there is a lack of love concepts that take into account etiological dimensions and links to psychopathological vicissitudes of love. This would allow understanding love relations from a more dynamic functional perspective.

Given the frequency with which difficulties in love relationships are linked with clinical complaints, it would be helpful to obtain a better empirical understanding not of love itself, but of the individual capacity to love (CTL), and to develop means of assessing the components of this construct. This would allow for an enhanced study of personality characteristics associated with difficulties in love relationships, as well as those characteristics which may be strengthened in order to enhance relational functioning. Although close relationships are indisputably associated with well-being, the mechanisms involved remain less well-understood (Feeney and Collins, 2015). Measurement of the CTL may also be useful for clinicians who, in the course of addressing their patients’ psychological concerns, encounter various manifestations of impairments in the area of committed love relations.

Research suggests that love-related aspects such as social contact, libido and sexual activity are reduced during episodes of psychiatric disorders (Mathew and Weinman, 1982; Davidson and Turnbull, 1986). For example, a poor quality of social relationships or the relationship with partner and family is independently related to an increased risk of depression (Mamun et al., 2009; Teo et al., 2013) and highly anxious persons experience more conflicts in relationships than non-anxious individuals (Campbell et al., 2005). While stable intimate bonds are associated with psychological health (Burman and Margolin, 1992), the incapacity to maintain close relationships is associated with emotional distress (Bloom et al., 1978; Simon and Marcussen, 1999). Also, a propensity to engage in casual sex or sexual activity in uncommitted relationships (referred to as sociosexual orientation), is a predictor of instability of romantic relationships (Simpson et al., 2004; Penke and Asendorpf, 2008). Similarly, love themes are frequently present in suicide notes among both genders (Canetto and Lester, 2002) and suicidal behavior is often associated with disappointed love relationships (Séguin et al., 2014; Andreoli et al., 2016).

The here proposed concept of CTL is based on an integrated psychoanalytic theory formulated in terms of object relations theory (Modell, 1963; Bergmann, 1971; Kernberg, 1974a,b, 1977, 2011; Garza-Guerrero, 2000; Gottlieb, 2002) and empirically-based relationship science. The concept involves multiple components and refers to the ability to engage in, invest in, and sustain a committed romantic love relationship (Kernberg, 2011a). These components reflect critical aspects of psychological development theorized to contribute to successful partner relationship involvement. Indeed, from a developmental perspective, the CTL may be regarded as a culmination of complex processes that begin in early caregiving relationships (Zayas et al., 2011; Fraley and Roisman, 2015) and continue to be shaped throughout childhood, adolescent, and early adult developmental experience (Collins and Sroufe, 1999).

In terms of Erikson’s model of psychosocial development, the basic trust established in early caregiving relationships may provide a foundation for similar feelings of security in adult romantic relationships (Erikson, 1963; Marcia, 2014). This is consistent with attachment theory and research regarding the role of early experience in later partnerships (Bowlby, 1969; Hazan and Shaver, 1987; Pittman et al., 2011; Zayas et al., 2011). Later childhood and adolescent developmental achievements – characterized as autonomy, initiative, and identity – contribute to the individual’s ability to invest in relationships that involve intermittent disappointments, compromises, and potential separations (Erikson, 1963; Beyers and Seiffge-Krenke, 2010; Marcia, 2014). In this way, the ability to tolerate loss and mourning allows for the management of potential affronts to the individual’s sense of self in the face of inevitable relationship ruptures. Those who are averse to psychological pain may struggle to fully invest in a gratifying and meaningful – though imperfect – love relationship.

As a function of personality development, the CTL is thought to undergo considerable intensification during Erikson’s stage of Intimacy versus Isolation. In the successful negotiation of this phase of development, typically occurring in the emerging adulthood period, the individual develops the ability to share life interests and goals with another person (Erikson, 1963). Intimacy is thus both an interpersonal process involving the interactions of two people (Reis and Shaver, 1988), and an individual intrapsychic developmental achievement that portends for the health of long-term relationships (Weinberger et al., 2008). In the context of committed romantic relationships, intimacy involves the prioritization of a partner’s needs, as well as the acceptance of one’s vulnerability towards and dependence upon the partner (Kernberg, 2011a). Such vulnerability and dependence is likely to be intermittently tested during conflicts, calling for an ability to forgive and to repair relationship ruptures in the interest of the greater good of the couple as a unit. The blending of two identities into a common relational identity is further symbolized in passionate sexual relations in which transient experiences of merger may occur (Kernberg, 2011b). Individuals who lack the ability to develop a sustained absorption in the interests and goals of another, whilst simultaneously preserving a robust sense of personal identity, are likely to encounter difficulties in committed love relationships.

Numerous psychoanalytic theorists have drawn attention to personality structures that are organized at least in part around the management of intimate relatedness and its implications for the individual’s emotional equilibrium (Sullivan, 1953; Balint, 1979; Guntrip, 1992; Kernberg, 1995; Akhtar, 2000). Some individuals, for example, though desiring a love relationship, may dread an imagined engulfment – a perceived loss of autonomy – should they invest deeply in an intimate partnership. Some individuals may experience the investment in another as a depletion of the self, preferring instead an unrestricted sexuality with relatively limited emotional commitment. For others, the interdependence of a close relationship may evoke anxieties about the individual’s vulnerability and acceptability to a partner, potentially stimulating controlling behaviors aimed at both influencing the partner and regulating the individual’s feelings of insecurity. In this way, the failure to acquire a mature capacity for intimate, mutually gratifying, and deeply committed relatedness may be associated with self-regulatory psychopathology.

Pathological narcissism, a personality syndrome involving deficits in and maladaptive mechanisms regarding the maintenance of self-image, is perhaps exemplary of psychological circumstances involving an impaired CTL (Kernberg, 1995, 2011a; Kealy and Ogrodniczuk, 2014). Indeed, individuals with high levels of pathological narcissism tend to report anxious attachment patterns and histories of unsatisfactory relationships (Kealy et al., 2015), as well as domineering, vindictive, and intrusive interpersonal behaviors (Ogrodniczuk et al., 2009).

The present study had three objectives. The first objective was to operationalize the theory driven construct of CTL by developing a psychometric tool, the Capacity to Love Inventory (CTL-I), for assessing CTL and confirming the results using samples from two different countries. The second objective was to test the scale’s convergent and divergent validity relative to other presumably related psychological concepts (narcissism, depression, relationship quality, and sociosexual orientation). The third objective was to closer examine associations between dimensions of the CTL and these related psychological concepts as a way to advance the construct validity of CTL-I.

## Materials and Methods

In order to avoid problems in operationalization of the CTL construct, the study concept was oriented on the unified theory of construct validity (Messick, 1995). In synthesizing psychoanalytic theories, clinical observations, and empirical science regarding the CTL, Kernberg (2011a) has furnished a conceptualization of several areas that represent critical potential impediments in the area of romantic relational functioning. The components identified by Kernberg (2011a) served as our guide in developing a psychometric scale capable of measuring the CTL and comprised: Falling in love (FIL), Interest in the other (INT), Basic trust (BTR), Forgiveness (FRG), Gratitude (GRT), Common ego ideal (CEI), Mature dependency, Permanence of sexual passion (PSP), and Loss and mourning (LOM). To reflect each of the domains, psychoanalytic literature referenced by Kernberg (2011a) and additional theories on CTL were incorporated (Modell, 1963; Bergmann, 1971; Kernberg, 1974a,b, 1977; Garza-Guerrero, 2000; Gottlieb, 2002; Kealy and Ogrodniczuk, 2014). Based on these theories, content validity of items was assured by a joint discussion between research group members at the Capacity to Love Research Lab, and 70 items were generated in English and German simultaneously (NK, VW, MCL, and VB) then translated into Polish language and back translated by native speakers (KJ and NK).

### Participants

The present study utilized four different samples for the development and testing of the CTL-I. *Sample 1* (Austrian sample) was recruited to permit determination of the factorial structure of the initial 70-item questionnaire. The sample consisted of 547 (82.1% females) full datasets (out of 942 subjects who started but dropped off during some point of assessment) aged 16 to 66 (*M* = 28.92, *SD* = 10.22). They were recruited by snowball sampling procedure within the social network of medical students at the Medical University of Vienna, their families and friends, and were invited to fill an online questionnaire (German language). *Sample 2* (in Poland) consisted of 240 participants (82.9% females) aged 18 to 50 (*M* = 23.24, *SD* = 4.21), mainly psychology students at University of Warsaw, who were contacted by email addressed to students of the department. No dropouts and missing values were reported. The Polish sample was used to confirm the structure of the CTL-I that was derived from Sample 1. *Sample 3* (in Austria) consisted of 174 full datasets (out of 233) subjects (58.6% females) aged 18 to 70 (*M* = 29.53, *SD* = 12.10) recruited with the intention of assessing construct validity with reference to pathological narcissism. The same recruiting procedure was applied to another independent *Sample 4* (*N* = 85, out of 125 approaching subjects in Austria), which was recruited to enable investigation of test–retest reliability of the confirmed scale structure based on Samples 1 and 2. The participants were asked to fill the questionnaire at baseline and 4 weeks later. In all studies a forced-item procedure was adopted which does not allow participants to proceed if items were left blank. In all studies participants were asked to refer to an ongoing relationship or in absence of such to the last significant relationship they had. The studies were conducted under the code of the Declaration of Helsinki and received a positive decision (1515/2013, 1179/2015, and 1184/2015) from the ethics committee at the Medical University of Vienna.

### Questionnaires

#### Capacity to Love Inventory (CTL-I)

The initial 70-items of the prospective questionnaire was applied in sample 1 (German translation) and the final reduced version with 41 items was used in sample 2 (polish translation), samples 3 (German) and 4 (German). The full item list of the final version is presented in **Table 1**. The items were rated on a four-point Likert scale ranging between 1 and 4.

**Table 1:** Factor loadings (residual variances) from confirmatory factor analysis and item statistics in Austrian (AT) and Polish (PL) samples.

	*Interest in the other (INT)*	AT				PL			
			
		Loading (variance)	*M*(*SD*)	Skewness	Kurtosis	Loading (variance)	*M*(*SD*)	Skewness	Kurtosis
1	It is important to me to know the life plan of my partner.	0.49(0.26)	3,66(0.58)	-1.65	2.52	0.34(0.27)	3.75(0.55)	-2.43	6.26
2	I share my life plans with my partner.	0.62(0.33)	3,42(0.73)	-1.07	0.52	0.50(0.60)	3.18(0.90)	-0.86	-0.13
3	I am joyful to share my partner’s success.	0.68(0.14)	3,70(0.51)	-1.48	1.27	0.69(0.16)	3.75(0.56)	-2.59	7.46
4	I feel enriched to see the personal growth and life experience of my partner.	0.64(0.18)	3,67(0.55)	-1.54	1.78	0.63(0.25)	3.62(0.64)	-1.63	2.24
5	When my partner is unhappy, I also feel sad.	0.48(0.25)	3,60(0.57)	-1.28	1.59	0.53(0.51)	3.24(0.85)	-0.89	0.03
6	I can be empathic with my partner and try to understand her/him.	0.50(0.26)	3,53(0.58)	-0.97	0.83	0.58(0.23)	3.59(0.59)	-1.35	2.11
7	I am often bored with my partner.^∗^	0.45(0.43)	3,37(0.73)	-1.03	0.77	0.50(0.42)	3.36(0.75)	-1.00	0.54
	***Basic trust (BTR)***								
8	I trust that my partner is empathic with me when necessary.	0.57(0.40)	3,21(0.77)	-0.89	0.64	0.75(0.33)	3.02(0.87)	-0.69	-0.08
9	My weaknesses, inner conflicts and problems are open to the other.	0.71(0.32)	3,14(0.8)	-0.69	0.03	0.46(0.56)	3.20(0.85)	-0.78	-0.21
10	I can express my feelings and needs to my partner openly.	0.78(0.25)	3,31(0.79)	-0.87	-0.07	0.76(0.22)	3.43(0.73)	-1.26	1.42
11	I can trust my partner that in situations of uncertainty and ambivalence she/he can be emotionally supportive.	0.69(0.33)	3,33(0.79)	-1.01	0.40	0.78(0.22)	3.46(0.75)	-1.46	1.91
12	I feel being honest to my partner.	0.72(0.20)	3,60(0.64)	-1.60	2.32	0.82(0.15)	3.56(0.68)	-1.59	2.29
13	I keep secrets from my partner.^∗^	0.57(0.38)	3,37(0.75)	-1.09	0.86	0.36(0.81)	2.73(0.97)	-0.47	-0.70
14	I can confess my weaknesses to my partner.	0.62(0.33)	3,33(0.74)	-0.91	0.43	0.56(0.34)	3.48(0.70)	-1.29	1.35
15	I sometimes feel that my relationships are limited.^∗^	0.49(0.70)	2,62(0.96)	-0.16	-0.91	0.38(0.89)	2.72(1.02)	-0.37	-0.95
16	I am comfortable with my partner and I usually feel safe in his/her company.	0.67(0.30)	3,45(0.73)	-1.29	1.32	0.70(0.18)	3.60(0.60)	-1.46	2.23
	***Gratitude (GRT)***								
17	I feel gratitude for the existence of my partner.	0.67(0.22)	3,63(0.63)	-1.77	3.13	0.72(0.26)	3.57(0.73)	-1.73	2.48
18	I feel gratitude for the love received.	0.71(0.19)	3,65(0.61)	-1.90	3.80	0.75(0.25)	3.51(0.77)	-1.51	1.55
19	When separated I still feel connected with the partner.	0.64(0.34)	3,44(0.76)	-1.38	1.62	0.66(0.25)	3.57(0.67)	-1.60	2.50
20	I accept that I need my partner.	0.54(0.54)	3,19(0.87)	-0.75	-0.38	0.76(0.23)	3.56(0.74)	-1.78	2.71
21	I like to convey comfort to my partner.	0.66(0.22)	3,62(0.62)	-1.61	2.46	0.53(0.35)	3.59(0.70)	-1.79	2.92
22	I like to take care of the other, when he/she needs my help.	0.66(0.24)	3,56(0.65)	-1.52	2.37	0.58(0.23)	3.65(0.59)	-1.72	3.12
23	I like to share responsibilities in our daily life in order to take pressure off my partner.	0.54(0.36)	3,32(0.71)	-0.86	0.56	0.37(0.69)	2.96(0.90)	-0.45	-0.65
	***Common ego ideal (CEI)***								
24	I am dedicated to my relationships.	0.60(0.32)	3,28(0.70)	-0.73	0.40	0.69(0.20)	3.61(0.61)	-1.55	2.35
25	We always try to work on our relationship.	0.61(0.35)	3,13(0.74)	-0.57	0.02	0.57(0.32)	3.28(0.69)	-0.67	.25
26	I respect the personality and essential values of my partner.	0.53(0.27)	3,51(0.61)	-0.98	0.68	0.59(0.24)	3.59(0.61)	-1.53	2.84
27	I love to watch my partner’s gestures and reactions.	0.55(0.29)	3,54(0.64)	-1.19	0.81	0.51(0.29)	3.65(0.63)	-1.88	3.49
28	I feel committed to our joint life.	0.71(0.24)	3,46(0.70)	-1.28	1.49	0.71(0.19)	3.68(0.62)	-2.05	4.16
29	I search for compromise solutions when conflicts and competing agendas arise.	0.41(0.31)	3,42(0.61)	-0.71	0.57	0.45(0.37)	3.39(0.68)	-1.16	1.89
30	I often tell my partner that I love him.	0.60(0.59)	3,13(0.96)	-0.83	-0.37	0.60(0.74)	3.12(1.07)	-0.88	-0.58
31	I feel deeply connected with my partner.	0.74(0.25)	3,37(0.75)	-0.99	0.37	0.78(0.21)	3.51(0.73)	-1.47	1.63
	***Permanence of sexual passion (PSP)***								
32	Sexual boredom arises in long-term relationships.^∗^	0.79(0.31)	2,69(0.92)	-0.21	-0.79	0.95(0.08)	3.24(0.93)	-1.06	0.14
33	The sexual desire diminishes throughout time.^∗^	0.89(0.18)	2,76(0.92)	-0.13	-0.94	0.81(0.27)	3.25(0.88)	-1.06	0.36
	***Loss and mourning (LOM)***								
34	It is hard for me to accept when a loved person is not able to respond to my love.^∗^	0.46(0.62)	3,10(0.89)	-0.67	-0.42	0.41(0.77)	1.76(0.97)	-1.12	0.16
35	When a relationship is over, I often blame my ex-partner.^∗^	0.57(0.67)	3,05(1,00)	-0.72	-0.62	0.40(0.84)	2.78(1.00)	0.32	-0.99
36	I sometimes have wishes for revenge when my partner dismisses me.^∗^	0.57(0.68)	3,18(1,00)	-0.94	-0.34	0.45(0.84)	3.03(1.03)	0.68	-0.77
37	I am often unwilling to accept the end of my relationships.^∗^	0.66(0.63)	2,74(1,07)	-0.24	-1.21	0.59(0.82)	2.59(1.12)	0.09	-1.36
38	I am often jealous.^∗^	0.49(0.68)	2,73(0.94)	-0.31	-0.78	0.55(0.80)	2.47(1.07)	0.09	-1.26
39	I have feelings of guilt after a separation.^∗^	0.45(0.79)	2,78(0.99)	-0.31	-0.97	0.50(0.70)	2.53(0.97)	0.06	-0.96
40	I sometimes devaluate myself if my partner abandoned me.^∗^	0.62(0.74)	2,61(1,10)	-0.12	-1.31	0.72(0.59)	2.47(1.11)	-0.11	-1.34
41	It is hard for me to move on after a relationship.^∗^	0.40(0.93)	2,43(1,05)	0.06	-1.20	0.33(0.99)	2.26(1.05)	-0.26	-1.15


*^∗^Reversed item; *n* = 547 for Austrian (AT) and *n* = 240 for Polish (PL) sample.*

#### Quality of Relationship Inventory (QRI)

The Quality of Relationship Inventory (QRI) (Pierce et al., 1997) in the German translation was used (Reiner et al., 2012). It is a self-report questionnaire consisting of 25 items that are evaluated on a four-point Likert scale ranging from 1 (not true) to 4 (almost always true). QRI has 25 items forming three dimensions: Support (seven items, e.g., ‘To what extent could you count on this person to help with a problem?’), Conflict (12 items, e.g., ‘How critical of you is this person?’), and Depth (6 items, e.g., ‘How much do you depend on this person?’). The internal consistency for the subscales was 0.84, 0.89, and 0.82 for the respective subscales in a representative German sample (Reiner et al., 2012). Higher scores in Support and Depth dimensions mean better quality of relationship, whereas higher scores in the Conflict are interpreted in terms of lower quality of relationship. The questionnaire was administered in sample 1.

#### Beck Depression Inventory (BDI-II-R)

Beck Depression Inventory (BDI-II) is a well-established measure of depressive traits (Beck et al., 1988). It allows to be used as a screening measure as well as a measure of severity of depression based on 21 items rated between 0 and 3. Its German translation by Hautzinger et al. (1994) yields satisfying internal consistencies as measured by Cronbach’s α ranging between 0.76 and 0.95 in clinical samples and between 0.73 and 0.92 in non-clinical samples. The questionnaire was administered in sample 1.

#### Revised Version of the Sociosexual Orientation Inventory (SOI-R)

Sociosexual Orientation Inventory (SOI-R) by Penke and Asendorpf (2008) in the Polish translation (Jankowski, 2016) was used to measure sociosexual orientation. The questionnaire was administered in sample 2. Higher scores in SOI-R indicated unrestricted sociosexuality, whereas lower scores indicated more restricted orientation. The scale used in the study has nine items with a five-point Likert scale response format. It allows for quantification of three facets of sociosexual orientation, i.e., behavior, attitude, desire, and a total score. Each of the three dimensions consists of three items. Sample questions are: behavior ‘With how many different partners have you had sex within the past 12 months?’; attitude ‘Sex without love is OK’; desire ‘In everyday life, how often do you have spontaneous fantasies about having sex with someone you have just met?’ Typically, scores of each scale are expressed as the average of scores obtained from adherent items, and the total score is an average of the scores for the three facets. This allows for comparisons between subscales and between subscales and total score, and produces values between 1 and 9 for each subscale and for the total score. Cronbach’s α in the present study were high for behavior (0.79), attitude (0.82), and desire (0.88), and the total score (0.87).

#### Pathological Narcissism Inventory (PNI)

The original Pathological Narcissism Inventory (PNI) is a 52-item self-report measure assessing seven dimensions of pathological narcissism including measures of narcissistic grandiosity (Entitlement Rage, Exploitativeness, Grandiose Fantasy, and Self-sacrificing Self-enhancement) and narcissistic vulnerability (Contingent Self-esteem, Hiding the Self, and Devaluing) (Pincus et al., 2009). The applied German version includes a translation of the original and two additional items constructed to extend the exploitative subscale based on DSM diagnostic criteria (Morf et al., 2017). In the German validation study, Cronbach alphas for the subscales ranged between 0.82 (SSSE) and 0.92 (CSE) with an alpha coefficient for the total scale of 0.94. The retest reliability for the subscales ranged from *r* = 0.75 (DEV and SSSE) to 0.87 (CSE and GF) and the reliability for the total score was 0.86 (Morf et al., 2017). The questionnaire was used in sample 3.

#### Narcissistic Personality Inventory (NPI)

The original Narcissistic Personality Inventory (NPI) is a 40-item self-report measure developed by Raskin and Terry (1988) also available in a 15-items version (Schütz et al., 2004) assessing two dimensions of narcissism (Leadership and Grandiosity) from a social-personality psychology perspective. Leadership represents the ability to lead groups and others, while Grandiosity describes features of personality such as feelings to be a special and unique person. Some research indicates that the NPI assesses adaptive characteristics of narcissism such as achievement motivation, self-esteem, emotional resilience, and extraversion rather than pathological features (Pincus et al., 2009). The applied German NPI-15 translation (Schütz et al., 2004) showed good Cronbach alphas for the subscales with 0.73 and 0.82 and a good test–retest reliability *r* = 0.86 (Schütz et al., 2004). Recently, the two-factor structure was re-examined in a representative German population, resulting in Cronbach alpha of 0.82 for the Leadership and an acceptable 0.69 for the Grandiosity subscale (Spangenberg et al., 2013). The questionnaire was used in sample 3.

### Statistical Analysis

The factor structure of the CTL-I was examined by maximum likelihood confirmatory factor analysis (CFA) and goodness of fit was established basing on Hu and Bentler (1998) two-index presentation strategy. Specifically, for complex models, as in the presented work, it is suggested to interfere on fit, based on standardized root mean square residual (*SRMR*) together with root-mean-square error of approximation (*RMSEA*) with cutoff values indicative for acceptable fit of around 0.80 or less for *SRMR* and around 0.60 or less for *RMSEA* (Hu and Bentler, 1998). We supplemented the above fit indices with Akaike information criterion (*AIC*) and Bayesian information criterion (*BIC*) allowing for comparison between competing models (lower the values represent a better fit). Associations with other scales were tested with Pearson correlation and differences between groups were checked using *t*-test. Internal consistency reliability was assessed by Cronbach alpha. The calculations were performed by IBM SPSS and AMOS (version 22.0). Significance levels were set at 0.05.

## Results

### Factor Analysis

At first, a 70-item, eight-factor model consisting of FIL, INT, BTR, FRG, GRT, CEI, PSP, LOM was tested in the Austrian sample (sample 1) using CFA, with factors allowed to correlate with each other. Fit indices were: χ^2^(2317) = 7026.8, χ^2^/degrees of freedom = 3.03, *SRMR* = 0.086, *RMSEA* = 0.061 (95% *CI*: 0.059; 0.063), *AIC* = 7362.8, *BIC* = 8085.9, thus they showed mediocre fit to the data due to *SRMR* exceeding the commonly acknowledged threshold of 0.080 for good fit.

To improve the model fit, we retained items with factor loadings greater than 0.40 and, next, examined internal consistency of each of the eight scales by means of Cronbach alpha. Only scales with internal consistency of at least 0.70 were retained. As a result, the scales FIL (all nine items) and FRG (all seven items) could not be retained, and 13 further items from other subscales dropped out due to too low factor loadings. The resulting six-factor model (with 41 items) was re-tested with CFA, with scales being allowed to correlate with each other. Fit indices were: χ^2^(764) = 2391.9, χ^2^/degrees of freedom = 3.13, *SRMR* = 0.060, *RMSEA* = 0.062 (95% *CI*: 0.060; 0.065), *AIC* = 2585.9, *BIC* = 3003.4. Thus, similarly to the previous model χ^2^/degrees of freedom and *RMSEA* were acceptable, and SRMR lowered below threshold value of 0.080. What is more, both AIC and BIC values were lower for the six-factor model. Consequently, in comparison to the initial eight-factor model, the six-factor model (model 2) consisting of INT, BTR, GRT, CEI, PSP, LOM was improved and accepted as the final one.

The next step was to retest the six-factor model (model 2) by CFA in an independent (Polish) sample 2. The results confirmed item loadings on the six-factors. The fit indices were: χ^2^(764) = 1482.3, χ^2^/degrees of freedom 1.94, *SRMR* = 0.070, *RMSEA* = 0.063 (95% *CI*: 0.058; 0.067) indicating acceptable fit, which was comparable to that observed in sample 1 (Austria). Factor loadings and Cronbach alphas for the scales for the final model in both samples are shown in **Tables 1**, **2**, respectively.

**Table 2:** Correlations among factor scores in Samples 1 and 2 (Austria/Poland).

	INT	BTR	GRT	CEI	PSP	LOM	*Age*
INT	–	0.70^∗∗∗^/0.69^∗∗∗^	0.80^∗∗∗^/0.84^∗∗∗^	0.80^∗∗∗^/0.94^∗∗∗^	0.40^∗∗∗^/0.25^∗∗^	0.11/-0.03	-0.09/0.01
BTR			0.75^∗∗∗^/0.69^∗∗∗^	0.75^∗∗∗^/0.77^∗∗∗^	0.34^∗∗∗^/0.22^∗∗^	0.25^∗∗∗^/-0.07	-0.08/0.00
GRT				0.91^∗∗∗^/0.90^∗∗∗^	0.39^∗∗∗^/0.30^∗∗∗^	0.04/-0.16	-0.15^∗∗∗^/0.01
CEI					0.49^∗∗∗^/0.32^∗∗∗^	0.06/-0.12	-0.12^∗∗^/0.01
PSP						0.15^∗∗^/0.17^∗^	-0.06/0.03
LOM						–	0.22^∗∗∗^/0.28^∗∗∗^
CTL-I total	0.54^∗∗∗^/0.76^∗∗∗^	0.63^∗∗∗^/69^∗∗∗^	0.75^∗∗∗^/74^∗∗∗^	0.79^∗∗∗^/80^∗∗∗^	0.69^∗∗∗^/66^∗∗∗^	0.40^∗∗∗^/0.28^∗∗∗^	-0.05/0.10
Cronbach alpha	0.73/0.72	0.86/0.83	0.81/0.80	0.81/0.82	0.83/0.87	0.75/0.72	–


*^∗∗∗^*p* < 0.001; ^∗∗^*p* < 0.01; ^∗^*p* < 0.05; *n* = 547 for Austrian sample 1 and *n* = 240 for Polish sample 2. CTL-I scales: INT, interest in the other; BTR, basic trust; GRT, gratitude; CEI, common ego ideal; PSP, permanence of sexual passion; LOM, loss and mourning.*

### Internal Reliability and Validity of CTL-I Subscales

As shown in **Table 2**, the internal consistency after item reduction in each of the six subscales was good and comparable in both samples 1 and 2. The corresponding Cronbach’s alpha values for the total scale were 0.90 and 0.88 respectively. The total alpha was further confirmed in sample 3 with 0.89 (subscale alpha range = 0.68 to 0.82). Similarly, in sample 4, alphas for the CTL total score at time 1 was 0.90 and 0.92 (*N* = 85) at time 2 and alpha values for the subscales at both time points ranged between 0.67 and 0.86.

All six subscales correlated consistently with each other, with moderate associations of the subscale ‘Loss and Mourning’ with the subscales ‘Basic Trust’ in Sample 1 and ‘Permanence of Sexual Passion’ as shown in **Table 2**. When Bonferroni correction is adopted to the correlational analyses and twenty one coefficients are considered, *p* level should equal 0.002 or less to be considered statistically significant. Using this conservative method associations of PSP with LOM in both samples and with INT and BTR in the Polish sample could be regarded as less firm.

Interestingly, age was only associated with the subscales ‘Gratitude’ and ‘Loss and Mourning’ (association with CEI in the Austrian sample would not survive the Bonferroni correction).

**Table 3** shows small but significant differences between the Austrian (1) and Polish (2) sample in most subscales. The gender comparison showed that males scored slightly higher than females on the subscale ‘*Loss and Mourning*’. Additionally, Austrian males showed a lower mean than females in the ‘*Interest for the other*’ subscale (see **Table 4**), but the *p*-value of this association exceeds the value of 0.008 imposed by the Bonferroni correction considering six *t*-tests, making the association less firm.

**Table 3:** Descriptive statistics for the studied measures and comparison of means between countries.

	Austria			Poland		
		
	Mean (*SD*)	Skewness	Kurtosis	Mean (*SD*)	Skewness	Kurtosis
Interest in the other (INT)^∗∗∗^	3.32 (0.32)	-1.25	2.30	3.50 (0.43)	-1.22	2.16
Basic trust (BTR)^∗∗∗^	3.04 (0.36)	-0.97	0.73	3.24 (0.52)	-0.96	0.68
Gratitude (GRT)	3.49 (0.48)	-1.39	2.73	3.49 (0.49)	-1.85	4.76
Common ego ideal (CEI)^∗∗∗^	3.35 (0.47)	-1.11	1.87	3.48 (0.47)	-1.66	4.45
Perm. of sexual passion (PSP)^∗∗∗^	2.72 (0.85)	-0.17	-0.73	3.25 (0.85)	-1.10	0.44
Loss and mourning (LOM)^∗∗∗^	2.68 (0.61)	-0.29	-0.47	2.49 (0.60)	0.07	-0.50
CTL-I total^∗∗∗^	18.60 (1.97)	-0.72	0.88	19.47 (2.16)	-0.91	1.01


*^∗∗∗^*p* < 0.001; *n* = 547 for Austrian sample 1 and *n* = 240 for Polish sample 2.*

**Table 4:** Gender differences in CTL-I scales in AT and PL.

	Austria		Poland	
		
	Women mean (*SD*)	Men mean (*SD*)	Women mean (*SD*)	Men mean (*SD*)
Interest in the other (INT)	3.33 (0.32)	3.25 (0.34)^∗^	3.52 (0.42)	3.40 (0.45)
Basic trust (BTR)	3.04 (0.36)	3.03 (0.39)	3.25 (0.52)	3.20 (0.56)
Gratitude (GRT)	3.49 (0.48)	3.47 (0.44)	3.50 (0.47)	3.41 (0.58)
Common ego ideal (CEI)	3.37 (0.47)	3.30 (0.45)	3.49 (0.46)	3.41 (0.53)
Permanence of sexual passion (PSP)	2.72 (0.85)	2.74 (0.83)	3.26 (0.85)	3.16 (0.88)
Loss and mourning (LOM)	2.64 (0.61)	2.81 (0.50)^∗∗^	2.41 (0.58)	2.84 (0.61)^∗∗∗^
CTL-I total	18.60 (1.92)	18.60 (1.98)	19.45 (2.17)	19.41 (2.10)


*^∗∗∗^*p* < 0.001; ^∗∗^*p* < 0.01; ^∗^*p* < 0.05. *n* = 547 for Austrian sample 1 and *n* = 240 for Polish sample 2.*

### Convergent and Divergent Validity

Validity was examined by correlations between the CTL-I subscales and other variables. We hypothesized a positive association between CTL and relationship quality in sample 1. Each of the six scales of the CTL-I was moderately correlated with each of the three dimensions of the QRI. According to expectations, the total CTL-I score and all scales of the CTL-I correlated positively with the dimensions Depth and Support and negatively with the Conflict dimension of QRI. The only exception was the CTL-I subscale ‘Loss and Mourning,’ which was not significantly correlated with the dimension Depth of QRI (see **Table 5**).

**Table 5:** Correlations between dimensions of capacity to love and quality of relationship inventory and depression scores (*M* = 9.13, *SD* = 8.59) (Austrian sample 1, *n* = 531).

	QRI support	QRI conflict	QRI depth	BDI
Interest in the other (INT)	0.49^∗∗∗^	-0.36^∗∗^	0.58^∗∗∗^	-0.19^∗∗^
Basic trust (BTR)	0.68^∗∗∗^	-0.51^∗∗∗^	0.62^∗∗∗^	-0.31^∗∗^
Gratitude (GRT)	0.54^∗∗∗^	-0.37^∗∗∗^	0.74^∗∗∗^	-0.19^∗∗^
Common ego ideal (CEI)	0.52^∗∗∗^	-0.37^∗∗∗^	0.67^∗∗∗^	-0.19^∗∗^
Permanence of sexual passion (PSP)	0.28^∗∗∗^	-0.26^∗∗∗^	0.32^∗∗∗^	-0.21^∗∗^
Loss and mourning (LOM)	0.21^∗∗∗^	-0.36^∗∗∗^	-0.01	-0.44^∗∗^
CTL-I total	0.63^∗∗∗^	-0.53^∗∗∗^	0.66^∗∗∗^	-0.37^∗∗^


*^∗∗∗^*p* < 0.001; ^∗∗^*p* < 0.01.*

In line with the hypothesis that depressive symptoms are associated with limitations to the CTL, all scales of CTL-I were inversely correlated with depression scores (BDI) (see **Table 5**).

We hypothesized that unrestricted SOI-R, which is a propensity to engage in casual sex or sexual activity in uncommitted relationships, would be negatively correlated with CTL. In fact, we found that the total SOI-R score was negatively correlated with five scales of CTL-I with the exception of ‘Loss and Mourning.’ This result seems to be based mainly on the correlation of the two dimensions of SOI-R *Attitude* and *Desire*. The third SOI-R dimension *Behavior* was not related to CTL-I subscales with the exception of the ‘Permanence of Sexual Passion’ scale (see **Table 6**).

**Table 6:** Correlations between the CTL and SOI-R scales (*n* = 240, Polish sample 2).

	SOI-R behavior	SOI-R attitude	SOI-R desire	SOI-R total score
Mean (SD)	1.86 (0.78)	2.60 (1.22)	2.39 (1.05)	2.38 (0.82)
Interest in the other (INT)	-0.10	-0.34^∗∗∗^	-0.34^∗∗∗^	-0.35^∗∗∗^
Basic trust (BTR)	-0.11	-0.27^∗∗∗^	-0.27^∗∗∗^	-0.28^∗∗∗^
Gratitude (GRT)	-0.04	-0.28^∗∗∗^	-0.29^∗∗∗^	-0.27^∗∗∗^
Common ego ideal (CEI)	-0.01	-0.29^∗∗∗^	-0.32^∗∗∗^	-0.28^∗∗∗^
Permanence of sexual passion (PSP)	-0.19^∗∗^	-0.27^∗∗∗^	-0.40^∗∗∗^	-0.37^∗∗∗^
Loss and mourning (LOM)	-0.02	0.04	-0.12	-0.04
CTL-I total	-0.14^∗^	-0.36^∗∗∗^	-0.46^∗∗∗^	-0.42^∗∗∗^


*^∗∗∗^*p* < 0.001; ^∗∗^*p* < 0.01; ^∗^*p* < 0.05.*

The association between CTL-I subscales and pathological narcissism (see **Table 7**) was examined within sample 3. In line with expectations, the total CTL-I and total PNI score were moderately and inversely correlated. The CTL-I subscales ‘Loss and Mourning’ as well as ‘Basic Trust’ contributed most to the association. On the other hand, the narcissistic aspects ‘Hiding the self,’ ‘Vulnerability,’ and ‘Devaluing’ contributed most to restrictions in CTL. As further expected, none of the NPI dimensions nor the total score was substantially associated with CTL-I total and subscales.

**Table 7:** Correlations between the CTL dimensions and narcissistic personality and pathological narcissism scores (*n* = 180, Austrian sample 3).

		INT	BTR	GRT	CEI	PSP	LOM	CTL-I total
PNI	CSE	0.03	-0.15*	-0.03	0.00	-0.15	-0.60**	-0.26**
	DEV	-0.09	-0.26**	-0.16*	-0.11	-0.08	-0.51**	-0.31**
	ER	-0.05	-0.17*	-0.11	-0.10	-0.16*	-0.45**	-0.28**
	EXP	-0.03	-0.01	-0.03	-0.04	-0.07	-0.08	-0.08
	GF	-0.03	-0.10	-0.04	-0.02	-0.01	-0.47**	-0.18*
	HS	-0.13	-0.30**	-0.23**	-0.20**	-0.16*	-0.47**	-0.38**
	SSSE	0.05	-0.05	0.04	0.06	0.00	-0.40**	-0.09
	GRAND	-0.02	-0.11	-0.05	-0.03	-0.07	-0.46**	-0.20**
	VULN	-0.05	-0.25**	-0.13	-0.09	-0.15*	-0.60**	-0.34**
	PNI total	-0.04	-0.19**	-0.10	-0.07	-0.12	-0.57**	-0.29**
								
NPI	LEAD	0.03	-0.10	0.13	0.09	-0.10	0.04	0.01
	GRAN	0.06	0.11	0.15*	0.16*	0.00	0.16*	0.15*
	NPI total	0.04	-0.04	0.16*	0.12	-0.08	0.09	0.05


*^∗∗^*p* < 0.01; ^∗^*p* < 0.05. PNI scales: CSE, Contingent Self-Esteem; DEV, Devaluing; ER, Entitlement Rage; EXP, Exploitativeness; GF, Grandiose Fantasy; HS, Hiding the Self; SSSE, Self-Sacrificing Self-Enhancement; GRAND, Narcissistic Grandiosity Subscale; VULN, Narcissistic Vulnerability Scale. NPI scales: LEAD, Leadership; GRAN, Grandiosity. CTL-I scales: INT, interest in the other; BTR, basic trust; GRT, gratitude; CEI, common ego ideal; PSP, permanence of sexual passion; LOM, loss and mourning.*

### Test–Retest Reliability

Within sample 4, the test–retest reliability for the total CTL-I score was r_tt_ = 0.81. The reliabilities for the subscales ranged from r_tt_ = 0.64 (GRT) to r_tt_ = 0.85 (LOM) (see **Table 8**).

**Table 8:** Test–retest correlations (*N* = 85, Austrian sample 4).

	INT 1	BTR 1	GRT 1	CEI 1	PSP 1	LOM 1	CTL total 1
INT 2	0.71^∗∗^	0.49^∗∗^	0.63^∗∗^	0.59^∗∗^	0.42^∗∗^	0.01	0.62^∗∗^
BTR 2	0.59^∗∗^	0.77^∗∗^	0.57^∗∗^	0.53^∗∗^	0.41^∗∗^	0.15	0.71^∗∗^
GRT 2	0.53^∗∗^	0.53^∗∗^	0.64^∗∗^	0.56^∗∗^	0.39^∗∗^	-0.03	0.58^∗∗^
CEI 2	0.59^∗∗^	0.60^∗∗^	0.60^∗∗^	0.73^∗∗^	0.50^∗∗^	0.05	0.70^∗∗^
PSP 2	0.33^∗∗^	0.40^∗∗^	0.31^∗∗^	0.43^∗∗^	0.81^∗∗^	0.01	0.48^∗∗^
LOM 2	0.08	0.27^∗^	-0.05	-0.01	-0.08	0.85**	0.40^∗∗^
CTL total 2	0.62^∗∗^	0.72^∗∗^	0.58^∗∗^	0.61^∗∗^	0.46^∗∗^	0.34**	0.81^∗∗^


*^∗∗^*p* < 0.01; ^∗^*p* < 0.05. CTL-I scales: INT, interest in the other; BTR, basic trust; GRT, gratitude; CEI, common ego ideal; PSP, permanence of sexual passion; LOM, loss and mourning.*

## Discussion

The aim of the study was the psychometric operationalization of the construct of CTL. The underlying theoretical basis of the construct was derived from an integrated psychoanalytic theory of the CTL, with emphasis on recent object relations theory which understands current interpersonal relations as linked to early childhood development (Modell, 1963; Bergmann, 1971; Kernberg, 1974a,b, 1977, 2011a; Gottlieb, 2002; Kealy and Ogrodniczuk, 2014). Rather than descriptively characterizing styles of loving, the theory of CTL refers to functioning in romantic committed relationships commonly referred to as love relationships. Accordingly, the theory is based on the assumption that various inhibitions of personality functioning result in current limitations to the CTL and thus in interpersonal difficulties.

### Factor Analysis

In order to test the factor structure of the instrument, a CFA approach was chosen because the coherent theoretical construct of CTL and its constituting dimensions were already defined within the framework of Kernberg (2011a). Out of an initial pool of 70 items constituting eight dimensions, the factor analysis finally confirmed 41 items in six dimensions: (1) Interest in the life project of the other, (2) Basic trust, (3) Humility and gratitude, (4) Common ego ideal, (5) Permanence of sexual passion, and (6) Acceptance of loss/jealousy/mourning. Only two initial dimensions ‘Falling in love’ and ‘Capacity for authentic forgiveness’ could not be confirmed as items needed to be excluded due to low loadings and unacceptable low internal consistencies of these subscales. In general, indices of CFA suggested an acceptable fit of the six-factor model and support the face validity of the concept. While all remaining six dimensions of the CTL-I showed moderate correlations with each other, the subscale ‘Acceptance of loss/Jealousy/Mourning’ represented by items like ‘*It is hard for me to accept if a loved person is not able to respond to my love*’ was only modestly related to ‘Basic trust’ and ‘Permanence of sexual passion.’ This scale represents coalesced aspects of reactions to loss of love objects, which are in line with the observed inverse associations with depression scores and conflicts in relationships. However, ‘Acceptance of loss/Jealousy/Mourning’ is not related to depth of the relationship or perceived support therein. It can therefore be seen as a rather stabilizing or neutralizing function in critical times rather than one that adds to depth or intensity of love relations.

### Validity

#### CTL and Symptoms of Depression

A framework of Freud’s comprehensive theory of depression has only recently been formulated for empirical testing and suggests close links between depression and loss of loved objects (Desmet, 2013). In line with our expectations, CTL is inversely associated with depressive symptoms in our study. The hypothesis has a long tradition among many theorists beyond Freud (1917) and his initial theory on the relation between loss of love objects and depression as a representation of the inability to mourn. In this sense, Balint (1952) described the difference between mature and primitive love as determined by strong narcissistic tendencies and unbearable depressive fears which may impair the ability to maintain loving relations. Thus, while loss and total unity with a love object are antagonistic extremes, mature CTL as the ability to bear depressive feelings (Klein, 1940, 1946) represents a protective feature against its symptomatic expressions at both ends of the continuum in form of depression and narcissism. More recent empirical results show that perceived parental love inconsistency is associated with later proneness to depression (Trumpeter et al., 2008). Conversely, depression in adolescence may impair subsequent romantic relationship qualities into late adolescence and emerging adulthood (Vujeva and Furman, 2011), corresponding to an impaired CTL. In the process of transition to parenthood, for example, love received from husband is seen by Benedek (1949, 1959) as a remedy for postpartum depression, which by restoring a narcissistic loss, in turn allows mother to be the source of love for the child. In this sense, further research might help to understand the early effects of parenting styles (Busch and Kapusta, unpublished) and the early nurturing co-parenting environment (Kapusta et al., 2017) on the development of the CTL in offspring.

#### CTL and Sociosexual Orientation

The concept of sociosexuality, which is related to promiscuity, describes individual differences in the readiness to engage in uncommitted sexual relationships (Penke and Asendorpf, 2008), and thus reflects the capacity to restrict one’s own sexual propensity to one love object. Restricted sociosexuality is also related to romantic relationship stability and quality (Simpson, 1987; Simpson and Gangestad, 1991, 1992; Ellis, 1998; Jones, 1998). In line with these facts, our results show that sociosexuality is inversely related to the CTL. The relevance of the ability to restrict one’s own sexual propensity seems to be reflected in the emotional and motivational aspects captured by the SOI, namely *attitudes* toward and *desire* for unrestricted sexuality. In contrast, the *behavior* dimension of the SOI, which counts sexual contacts and describes the lifetime allocation of effort to short-term versus long-term mating tactics, was only marginally related to CTL in our Polish sample. The *behavior* dimension was inversely correlated with the CTL scale ‘*Permanence of sexual passion*’. It seems possible that the sociosexual behavior of our rather young sample does not adequately differentiate between immature and mature CTL, given that sociosexuality increases with age (Penke and Asendorpf, 2008; Jankowski, 2016), and the rather low mean of the SOI *behavior* dimension in our sample reflects low sexual experience relative to other comparable studies (Penke and Asendorpf, 2008; Jankowski, 2016).

The gender comparison showed that males in both samples 1 and 2 scored higher than females on the subscale ‘*Loss and Mourning*’ and Austrian males showed a lower ‘*Interest for the life plan of the other*’ than females (**Table 4**). The mean scores of the CTL-I subscales were similarly high in both the Polish and Austrian samples, with the exception of higher means of PSP among Polish participants. It remains to be elucidated in the future, whether this difference between countries is based on cultural/religious, sampling or linguistic differences (**Table 3**). Although the demonstration of cultural differences between Austria and Poland is beyond the scope of this work, for example, religious beliefs differ between Austria and Poland considerably, with Poland exhibiting more religiousness (Coutinho, 2016). Given the fact that ‘*Permanence of sexual passion*’ is inversely related to promiscuity as measured by SOI-R, our results are supported by the argumentation that *Permanence of sexual passion* is disclosed at higher levels in a more religious country. However, we also admit that the PSP scale could be improved in future as it consists of only two items in the final 41-item CTL-I version.

#### CTL and Quality of Relationships

Since the functions of the CTL are experienced within relationships, we hypothesized a positive association between CTL and relationship quality. The QRI measuring the dimensions of *support*, *conflict*, and *depth* of relationships is based on theoretical models of social *support* which include interpersonal, intrapersonal, and situational efforts of exchange between two participants (Pierce et al., 1991, 1997). The QRI is based on the assumption that general predispositions to engage in and respond to social behavior are grounded in expectations, derived from Bowlby’s (1980) theory of working models and relations between the self and important others. Perceived qualities of *depth* and *support* in intimate relationships were associated most strongly with the domains *BTR*, *gratitude* and a *common ego ideal* of the CTL-I. In applying Mikulincer and Shaver’s (2005) model of the interplay between the caregiving and the attachment behavioral systems, in which one person responds to signals of need emitted by the other, the reduction of another person’s suffering by provision of support or experience of positive emotions fosters the experience of gratitude and may strengthen attachment security and *BTR*. Also, a perception of a shared ego ideal may increase mutual understanding and thus increase feelings of depth in a relationship. The opposite is true for *conflict* in intimate relationships, which was inversely related to all CTL-I subscales, and notably reflects a loss of *BTR*, reduced feelings of *gratitude* and restrictions in *common ego ideal*. These results are in line with considerations of the emergence of relational tensions and conflicts in the presence of a malfunctioning of the attachment and caregiving system, which otherwise tend toward a maintenance of stable and mutually satisfactory affectional bonds (Mikulincer and Shaver, 2008). Future studies should assess the associations between CTL-I and attachment styles, the latter being likely a function of the mature dependency dimension of CTL-I, and test the hypothesis formulated by Hazan and Shaver (1987) who argued for understanding romantic love as an attachment process.

#### Pathological Narcissism and Limitations to the Capacity to Love

Theoretical assumptions suggest that pathological narcissism is associated with an overall difficulty in the CTL or as a fundamental impairment of it (Gottlieb, 2002; Kernberg, 2011a; Kealy and Ogrodniczuk, 2014). In contrast to pathological narcissism with its incapability to love others, balanced love for oneself is generally held to be an essential component of healthy psychosocial functioning (Kealy and Ogrodniczuk, 2014). Pincus et al. (2009) convincingly show that the two measures of narcissism, namely PNI and NPI assess different aspects of narcissism, with the latter capturing more adaptive expressions of healthier and extraverted narcissistic features.

According to our expectations, CTL was moderately and negatively associated with pathological narcissism in our study, but not with the adaptive narcissism as measured by NPI. Interestingly, it was the vulnerability of narcissistic persons that was associated with limitations in the CTL, most notably the CTL-I domains of *BTR* and *LOM*. This is in line with theory, which points to problematic mourning processes in the context of separation from loved objects, due to lack of object constancy. According to Kernberg (2010), instead of mourning, persons with pathological narcissism blame others for the loss of the loved object, and in this way inhibit (and are thus protected from) the painful mourning process. This is often accompanied by a denial that the object could have his or her own independent existence, or in a more omnipotent processing, narcissistic personalities deny their own dependence on others (Garza-Guerrero, 2000).

#### Falling in Love and Authentic Forgiveness

Some limitations of our approach need to be discussed. Due to poor psychometric properties, the two initial dimensions of “*falling in love*” and “authentic forgiveness” were dropped from the final version of the CTL-I. It is not possible to evaluate if this rather reflects problems of the conceptualization and operationalization of these dimensions in the assessment instrument or if these dimensions are indeed no central prerequisites of the CTL. Some authors argue that falling in love represents a process of idealization which can lead to both successful and frustrating experiences in relationships depending on the level of maturity of the idealization process itself, which means that normal vs. pathological idealizations need to be differentiated (Kernberg, 1976, p. 191ff; Garza-Guerrero, 2000). We believe that our attempt to conceptualize the ‘falling in love’ dimension with items like ‘*I have experienced falling in love in my life often*’ or ‘*It often happens that I idealize my partner*’ may not have sufficiently captured the theoretically ambiguous concept of falling in love. The role of falling in love in mature loving remains unclear and although falling in love seems related, it not necessarily is a characteristic of the capacity for mature love (Kernberg, 1976, p. 237; Kernberg, 2011a). Future attempts to conceptualize falling in love should take non-pathological aspects of idealization into account.

Similarly, the factor analysis could not confirm the dimension ‘*Capacity for authentic forgiveness*’. Kernberg’s (2011a) understanding of authentic forgiveness is based on the acknowledgment of one’s own aggressive potential, the experience of trust and the communication of feelings of being hurt without blaming. We attempted to operationalize these aspects initially in the 70-item version of the CTL-I with items like ‘*When feeling misunderstood or hurt, I express my feelings to the other*’ or the inverse statement ‘*When hurt, I often try to induce guilt feelings in my partner.*’ However, the items did not integrate into a consistent forgiveness scale as expected. Future attempts at operationalizing authentic forgiveness should try to broaden the concept by including other salient facets such as the empathy for the offending partner’s motives (McCullough et al., 1998; Akhtar, 2013, p. 130), which has been linked to the mentalizing capacity of an individual Fonagy (2009, p. 447).

## Conclusion

According to the objectives of the study, (1) we were largely able to empirically confirm the concept of CTL by operationalization of its theoretical assumptions and have demonstrated that the 41-item CTL-I yields good internal consistency with stable and consistent results in two culturally different samples, and very good test–retest reliability. (2) The scale’s convergent and divergent validity has coherently been established in relation to narcissism, depression, relationship quality and sociosexual orientation. (3) A closer examination of the associations between dimensions of the CTL suggests a further refinement need for the dimension of ‘permanence of sexual passion’ to improve the construct validity of CTL-I. Also, the dimensions ‘*Falling in love*’ and ‘*Forgiveness*’ which could be not confirmed by means of CFA in this work should be re-aproached in future.

The so established CTL-I allows self-assessment and empirical testing of the relation between CTL-I and other concepts in future, thereby contributing to further understanding of the construct of CTL. Such an instrument might be suitable for the measurement of changes in psychoanalytic and other psychotherapeutic interventions and to help psychotherapists to understand their patients limitations and resources with respect to relationship issues. The resulting CTL-I also adds to the strong need for operationalization of psychoanalytic concepts to promote further empirical studies in psychoanalysis.

## Author Contributions

NK has developed the project to operationalize the concept of CTL, conceptualized all study designs 1, 2, 3, 4 and serves also the group leader of the CTL research lab. NK has written the manuscript draft and coordinated all researchers contributions in this project. KJ calculated statistics for studies 1 and 2 (CFA), NK for studies 3 and 4. The following authors contributed to the design and performed the following studies, VW, NK, VB, and MG study 1, NK, VB, and KJ study 2, CH, NK, and AS study 3, ML, JO, DK, and NK study 4. All authors reviewed the manuscript draft, contributed to manuscript writing and literature search and confirmed the manuscript submission.

## Conflict of Interest Statement

The authors declare that the research was conducted in the absence of any commercial or financial relationships that could be construed as a potential conflict of interest.
